# The complete mitochondrial genome of an important medicinal plant, *Rehmannia glutinosa* (Gaertn.) DC., 1845 (Lamiales, Orobanchaceae)

**DOI:** 10.1080/23802359.2024.2444611

**Published:** 2024-12-21

**Authors:** Yu Bai, Huan An, Rengang Zhang, Yanna Ma, Hongjia Zhang, Zhili Guo, Li Zhao, Zhaoxuan Wang

**Affiliations:** a Shijiazhuang Medical College, Shijiazhuang, China; b Shijiazhuang People’s Medical College, Shijiazhuang, China; c Yunnan Key Laboratory for Integrative Conservation of Plant Species with Extremely Small Populations/State Key Laboratory of Plant Diversity and Specialty Crops, Kunming Institute of Botany, Chinese Academy of Sciences, Kunming, China; d Bureau of Retired Officials of Huancui District, Weihai, China; e National Key Laboratory of Non-food Biomass Energy Technology, Nutrition and Health Research Institute, China Oil Foodstuffs Corporation (COFCO), Beijing, China; f College of Life Science and Technology, Beijing University of Chemical Technology, Beijing, China; g Department of Ecology and Environmental Science, Umeå University, Umeå, Sweden

**Keywords:** Mitogenome, phylogeny, *Rehmannia glutinosa*

## Abstract

*Rehmannia glutinosa*, an extensively utilized Chinese herbal medicine, is highly valued for its medicinal properties. In this study, the complete mitochondrial genome (mitogenome) of *R. glutinosa* was sequenced and assembled for the first time. The mitogenome is 547,032 bp in length, with an overall GC content of 44.97%. The mitogenome contains 67 unique genes, comprising 43 protein-coding, three rRNA, and 21 tRNA genes, with six protein-coding and nine tRNA genes being chloroplast-derived. The phylogenetic analysis, based on the maximum-likelihood criterion, demonstrated that *R. glutinosa* is closely related to *Aeginetia indica* and *Castilleja paramensis* within the family Orobanchaceae.

## Introduction

*Rehmannia glutinosa* (Gaertn.) DC., 1845 (Chinese foxglove) is a perennial herb with an exceptionally high medicinal value in traditional Chinese medicine. It is widely distributed and has been utilized as a folk medicine in China for thousands of years (Zhang et al. 2008). Modern pharmacological studies have demonstrated that *R. glutinosa* and its active principles possess wide pharmacological actions (anti-tumor and anti-senescence properties, etc.) on the blood system, immune system, endocrine system, cardiovascular system, and nervous system (Zhang et al. 2008). Moreover, *R. glutinosa* is a non-parasitic plant belonging to the family Orobanchaceae encompassing a full range of trophic specialization, including non-parasitic, hemiparasitic, and holoparasitic lineages (Li et al. 2019). Consequently, elucidating the phylogenetic position of *R. glutinosa* within the Orobanchaceae and exploring its genetic relationships with other members of this family is crucial for advancing our understanding of the evolutionary dynamics within this taxonomic group. The outcomes of this study are expected to have substantial implications for the molecular systematics of *R. glutinosa*, conservation of genetic diversity, and the rational utilization of medicinal plant resources within this genus.

## Materials and methods

**Figure 1.**
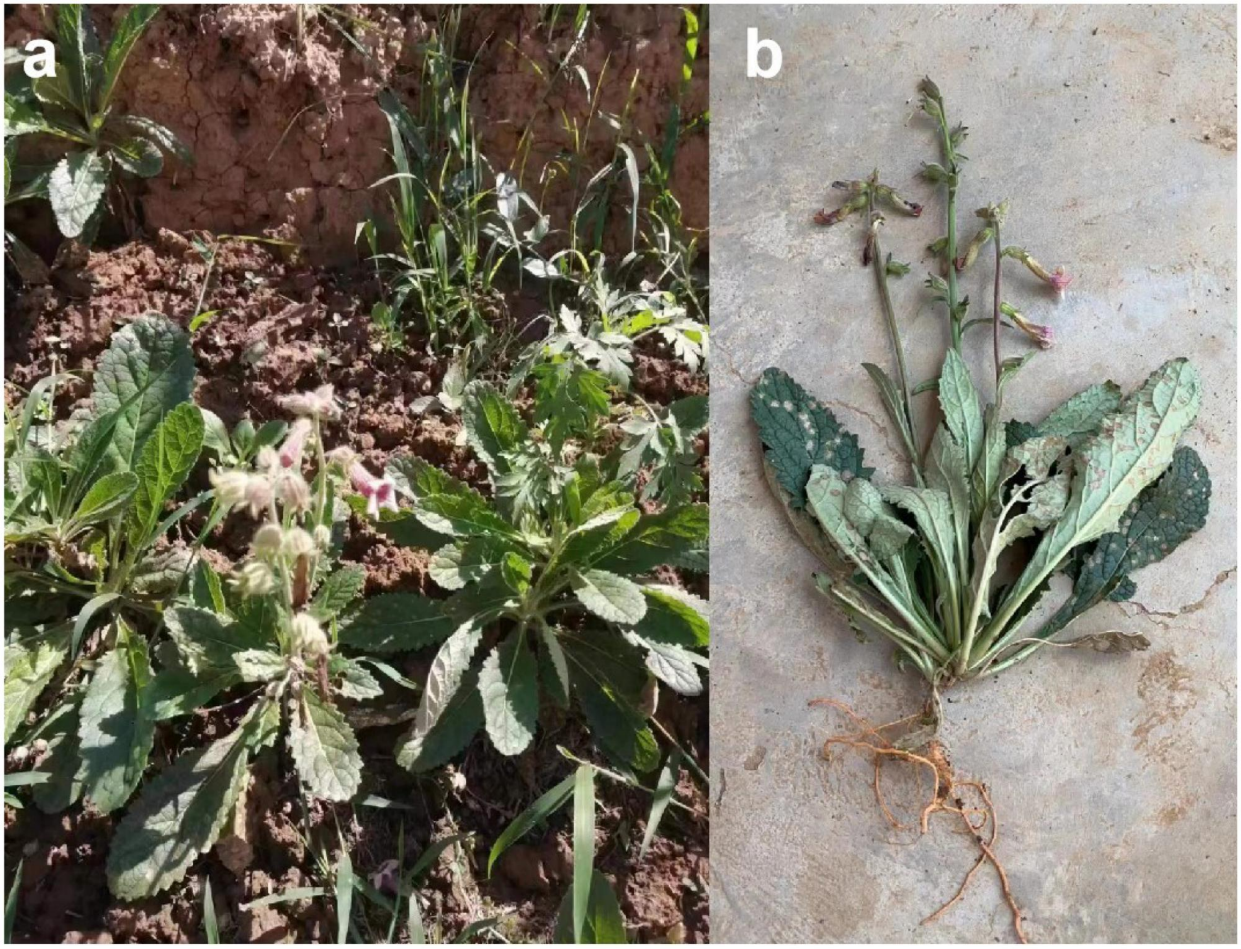
Morphology characteristics of *R. glutinosa* during flowering. (a) Basal leaves are usually rosulate. Stem leaves gradually decrease in size or are reduced to bracts upwards; leaf blades are ovate to narrowly elliptic; margins are irregularly crenate or obtusely serrate to toothed. Flowers are axillary or in terminal racemes. Pedicels are slender and ascending. (b) The fresh roots of *R. glutinosa* are yellow and can be used in traditional Chinese medicine. Photographs of *R. glutinosa* were taken by Huan An in Yuanshi County, Hebei Province, China (N 37.73°, E 114.51°).

The sample of *R. glutinosa* was collected from Yuanshi County, Shijiazhuang City, Hebei Province, China (N 37.73°, E 114.51°) (Figure 1, Figure S1). A specimen was deposited at the Herbarium (PE), Institute of Botany, Chinese Academy of Sciences (http://pe.ibcas.ac.cn/, Zhirong Yang and zry@ibcas.ac.cn) under the voucher number WCX001.

Genomic DNA was extracted from leaf materials using a modified CTAB method and the quality was assessed using the Qubit 2.0 system. A short-insert pair-end (2 × 150 bp) library was constructed using Illumina TruSeq DNA sample prep kit and sequenced on an Illumina HiSeq X Ten platform (Illumina Inc., San Diego, CA). The reads were filtered using Fastp software (Chen et al. 2018). The mitochondrial genome was assembled *de novo* using GetOrganelle v1.6.2e (Jin et al. 2020). For assembly validation, Oxford Nanopore Technologies (ONT) long reads were sequenced additionally and mapped to the assembly with minimap2 (Li 2018), and then the alignments were manually checked with the Integrative Genomics Viewer (Robinson et al. 2011). Native mitochondrial and chloroplast-derived genes were annotated using the OGAP (https://github.com/zhangrengang/OGAP) pipeline. The gene annotations were refined manually using Exonerate (Slater and Birney 2005). The gene map was constructed using the OGDRAW web server (https://chlorobox.mpimp-golm.mpg.de/OGDraw.html) (Greiner et al. 2019). Additionally, the entire *R. glutinosa* mitochondrial genome sequence was submitted to GenBank.

To ascertain the phylogenetic position of *R. glutinosa*, the complete mitochondrial genomes of other strains/species in the Lamiales were obtained from GenBank. A total of 37 mitochondrial protein-coding genes were subjected to alignment with MAFFT (Katoh and Standley 2013) and the multiple alignments were trimmed by TrimAl (Capella-Gutiérrez et al. 2009) with a parameter of ‘-automated1’. These trimmed alignments of each gene were concatenated into a single alignment of 28,125 bp. This alignment was used to construct a phylogenetic tree using on the maximum-likelihood (ML) criterion using IQ-TREE (Nguyen et al. 2015), with the best-fit model of GTR + F + R3 and bootstrapping with 1000 replicates (Hoang et al. 2018). *Solanum lycopersicum* was used as an outgroup.

## Results

**Figure 2.**
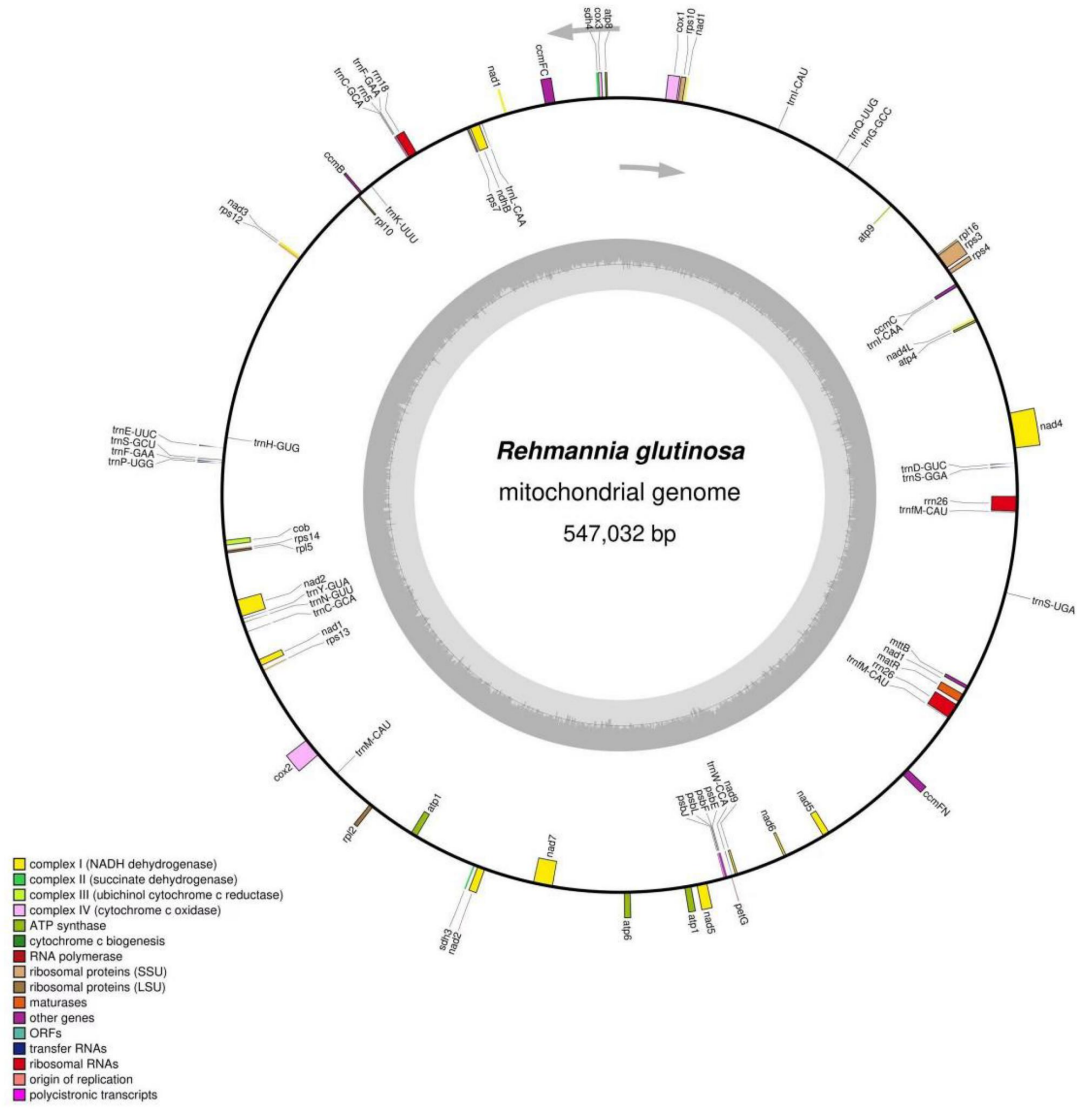
The gene map for the complete mitochondrial genome of *R. glutinosa*. Genes in the inner circle are transcribed clockwise, while those in the outer circle are transcribed counterclockwise. Different functional groups of genes are color coded. Darker gray shading represents DNA G + C content, while the lighter gray corresponds to A + T content. The functional classification appears in the bottom left corner.

The complete mitogenome of *R. glutinosa* (GenBank accession no. OM397952) is a single circular molecule, with a size of 547,032 bp (Figure 2; Figure S2). The overall GC content is 44.97%. Seven chloroplast-derived segments disperses in the mitogenome, with lengths ranging from 128 to 4403 bp and a total length of 10,477 bp. The mitogenome contains 67 unique genes, including 37 native mitochondrial and six chloroplast-derived protein-coding genes, 12 native mitochondrial and nine chloroplast-derived tRNAs, and three rRNAs. Additionally, there are eight cis-splicing genes (*nad*4, *rps*3, *rps*10, *cox*1, *ccm*FC, *ndh*B, *cox*2, and *nad*7) and three trans-splicing genes (*nad*1, *nad*2, and *nad*5) (Figures S3 and S4).

**Figure 3.**
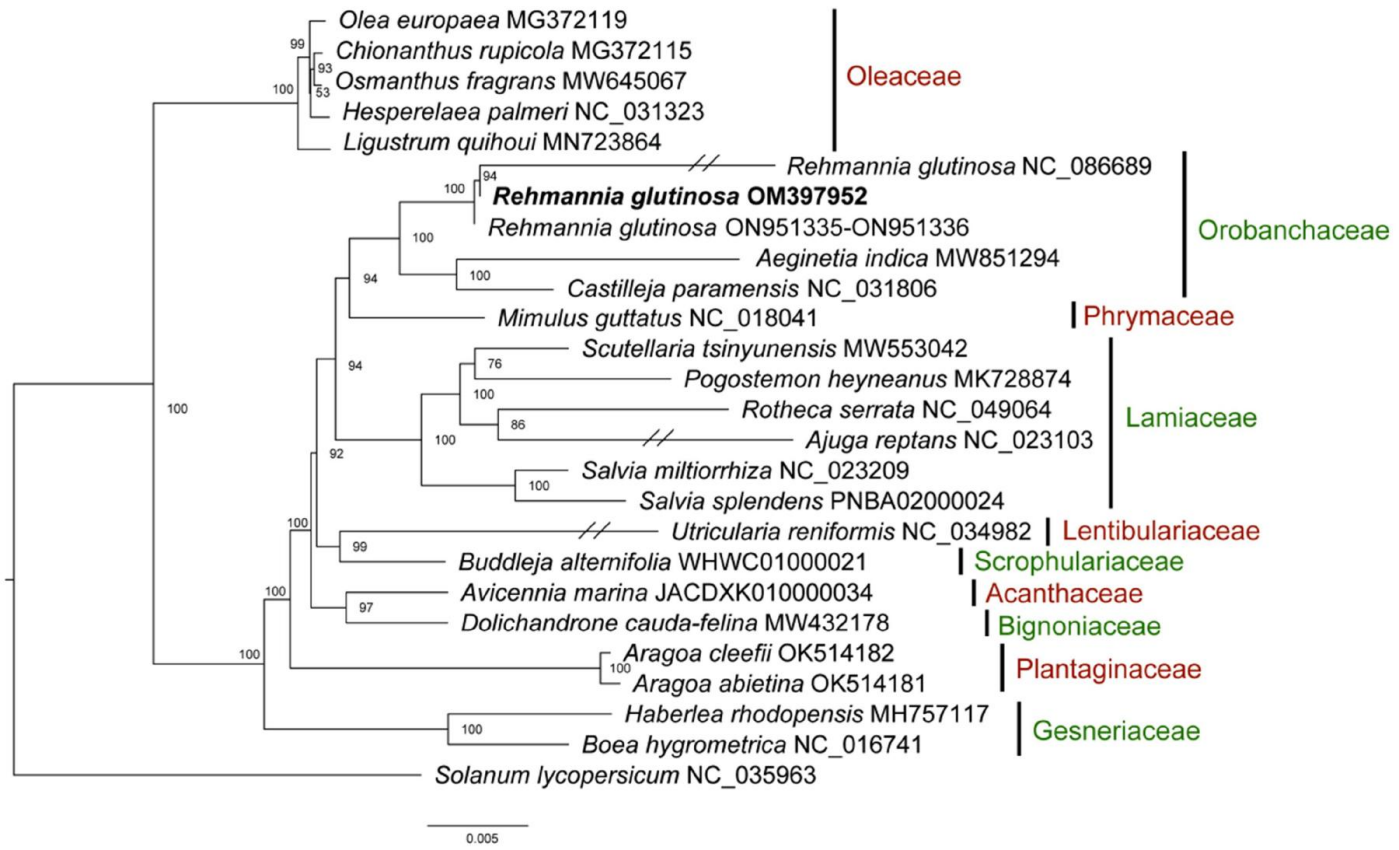
Phylogenetic tree includes *R. glutinosa* and its 23 relatives, highlighting the position of *R. glutinosa* (OM397952) in bold. A maximum-likelihood method was used to conduct the tree from a concatenated alignment of 37 mitochondrial protein-coding genes using GTR + F + R3 model with 1000 bootstrap replicates. The following sequences were used: *Rehmannia glutinosa* ON951335–ON951336 (Zeng et al. 2024), *Rehmannia glutinosa* NC_086689, *Olea europaea* MG372119 (Van de Paer et al. 2018), *Chionanthus rupicola* MG372115 (Van de Paer et al. 2018), *Osmanthus fragrans* MW645067 (Wang and Zhang 2021), *Hesperelaea palmeri* NC_031323 (Van de Paer et al. 2016), *Ligustrum quihoui* MN723864, *Aeginetia indica* MW851294 (Choi and Park 2021), *Castilleja paramensis* NC_031806, *Mimulus guttatus* NC_018041 (Mower et al. 2012), *Scutellaria tsinyunensis* MW553042 (Li et al. 2021), *Pogostemon heyneanus* MK728874, *Rotheca serrata* NC_049064, *Ajuga reptans* NC_023103 (Zhu et al. 2014), *Salvia miltiorrhiza* NC_023209, *Salvia splendens* PNBA02000024 (Jia et al. 2021), *Utricularia reniformis* NC_034982 (Silva et al. 2017), *Buddleja alternifolia* WHWC01000021 (Ma et al. 2021), *Avicennia marina* JACDXK010000034, *Dolichandrone cauda-felina* MW432178, *Aragoa cleefii* OK514182 (Mower et al. 2021), *Aragoa abietina* OK514181 (Mower et al. 2021), *Haberlea rhodopensis* MH757117, *Boea hygrometrica* NC_016741 (Zhang et al. 2011), and *Solanum lycopersicum* NC_035963. *Solanum lycopersicum* (Solanales) was set as the outgroup. The branches of *Ajuga reptans* and *Utricularia reniformis* were truncated since they were too long. Bootstrap support percent values are given at the nodes. Bar, 0.005 substitutions per site.

The phylogenetic analysis reveals that *R. glutinosa* is closely related to *A. indica* and *C. paramensis*, forming a monophyletic clade indicative of the family Orobanchaceae (Figure 3). The analysis demonstrated the family Orobanchaceae is sister to the family Phrymaceae, and the clade of Orobanchaceae + Phrymaceae is sister to the family Lamiaceae. It is inconsistent with a previous mitogenome-based study where the Orobanchaceae was sister to the Lamiaceae (Wang and Zhang 2021). The phylogenetic incongruence could result from the increased taxon sampling or possible incomplete lineage sorting. However, the other phylogenetic relationships within the order Lamiales are consistent with the previous study (Wang and Zhang 2021), such as the position of the family Oleaceae.

## Discussion and conclusions

This research offers an analysis of the *R. glutinosa* mitochondrial genome. The phylogenetic analysis reveals that *R. glutinosa* is closely related to *A. indica* and *C. paramensis*. The mitochondrial genome size of *R. glutinosa* was 547,032 bp, larger than those of *C. paramensis* (495,499 bp) and *A. indica* (401,628 bp). This total size of chloroplast-derived segments in *R. glutinosa* mitogenome was 10,477 bp, smaller than that in *C. paramensis* (79,336 bp) (Fan et al. 2016) but larger than that in *A. indica* (1205 bp) (Choi and Park 2021). Compared to the non-parasitic *R. glutinosa*, two genes (*sdh*4 and *rpl*2) in hemiparasitic *C. paramensis* are pseudogenized as determined previously (Fan et al. 2016) and three genes (*sdh*3, *sdh*4, and *rps*7) in holoparasitic *A. indica* are lost (Choi and Park 2021). It is worth noting that the *sdh4* became nonfunctional in both hemiparasitic plant *C. paramensis* and holoparasitic plant *A. indica* (Fan et al. 2016; Choi and Park 2021), while *R. glutinosa*, a non-parasitic plant, does not show this phenomenon. Whether the mitogenomic degradations of *C. paramensis* and *A. indica* are associated with their parasitic lifestyles remains to be further studied.

In NCBI, there are two additional complete mitochondrial genomes of *R. glutinosa*: ON951335–ON951336 (Zeng et al. 2024) and NC_086689 (Unpublished). Through a simple comparison, we found that the mitogenome ON951335.–ON951336 consists of two chromosomes, with a total size of 545,523 bp (chromosome 1: 497,303 bp, chromosome 2: 48,220 bp). However, the complete mitogenome of *R. glutinosa* in this study (GenBank accession no. OM397952) is a single circular molecule, with a larger size of 547,032 bp, which is also 1703 bp longer than that of NC_086689 (545,329 bp). Furthermore, the submission and release of the mitochondrial sequences in our study predated the other two genomes in GenBank.

The study of plant mitochondrial genomes facilitates the development of targeted strategies for plant improvement. Our findings provide clues for molecular breeding efforts for *R. glutinosa* and other plant species. Furthermore, this analysis of the organelle genomes advances our understanding of the Orobanchaceae mitogenome structure and evolution.

## Supplementary Material

（The clean copy）The complete mitochondrial genome of an important medicinal plant Rehmannia glutinosa.docx

（The clean copy）Supplementary Figures.docx

（The clen copy）Figure.docx

## Data Availability

The genome sequence data that support the findings of this study are openly available in GenBank of NCBI at https://www.ncbi.nlm.nih.gov/ under the accession no. OM397952. The associated BioProject, SRA, and BioSample numbers are PRJNA804417, SRR17933274 and SRR21395913, and SAMN25749585, respectively.
