# Supplementary material for: The complete mitochondrial genome of an important medicinal plant, Rehmannia glutinosa (Gaertn.) DC., 1845 (Lamiales, Orobanchaceae)
Source: Mitochondrial DNA B Resour. 2024 Dec 21;10(1):21–5. doi: 10.1080/23802359.2024.2444611 (PMC11703138; doi:10.1080/23802359.2024.2444611)
Supplement: （The clean copy）Supplementary Figures.docx [file TMDN_A_2444611_SM2892.docx]

**Supplementary Figures and Captions**


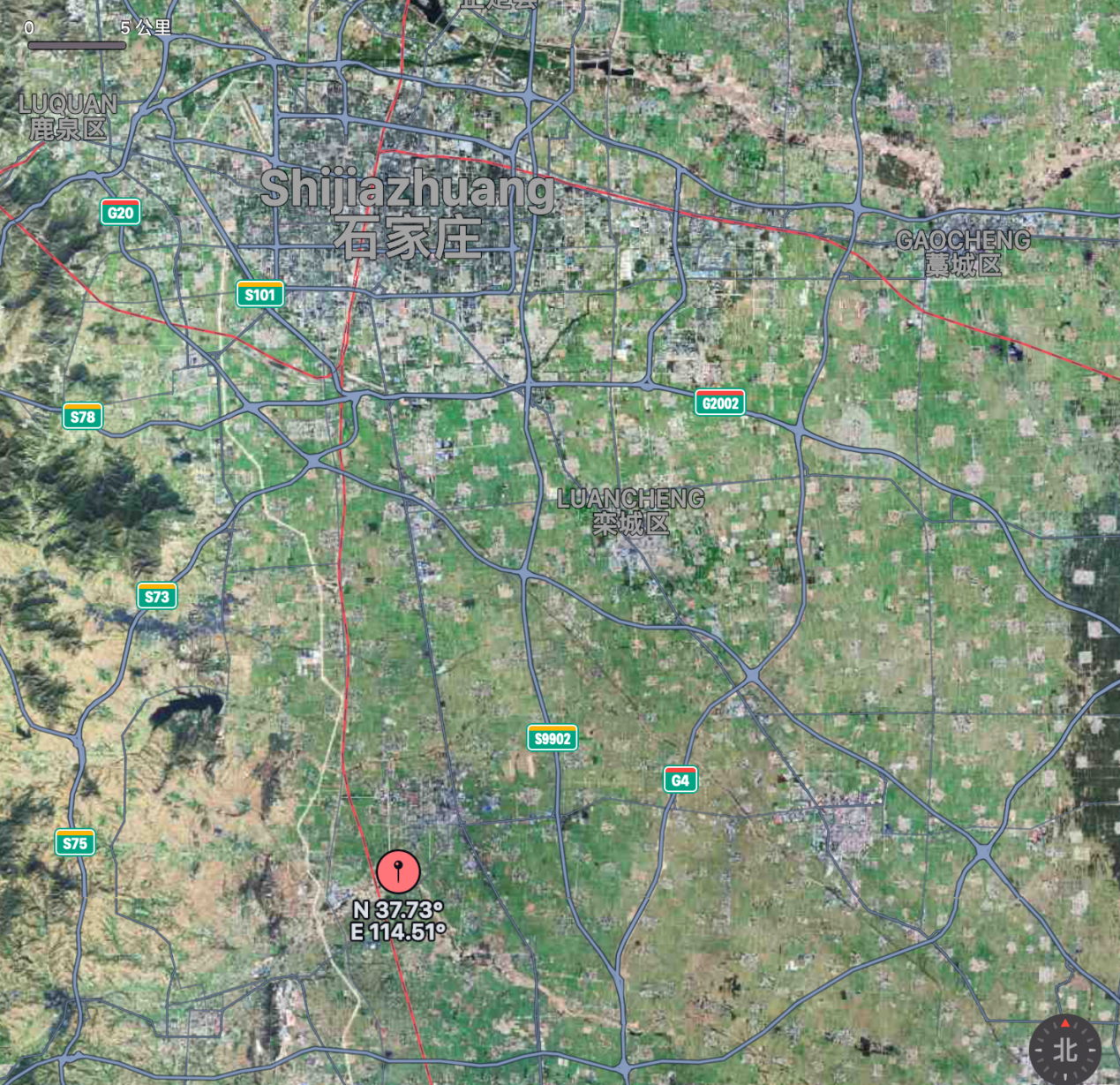


Figure S1. The map of sampling site. The sample of *Rehmannia glutinosa* was collected from Yuanshi County, Shijiazhuang City, Hebei Province, China (N 37.73°, E 114.51°).


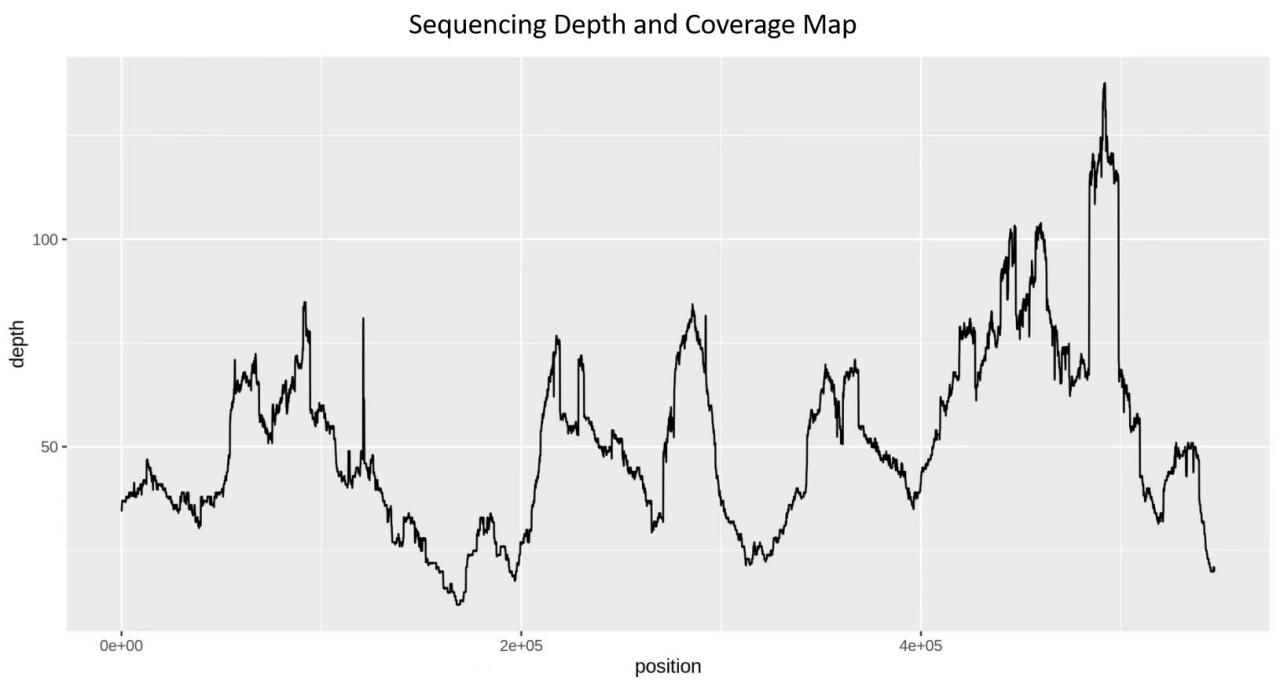


Figure S2. Read coverage depth map of the assembled genome of *Rehmannia glutinosa*.


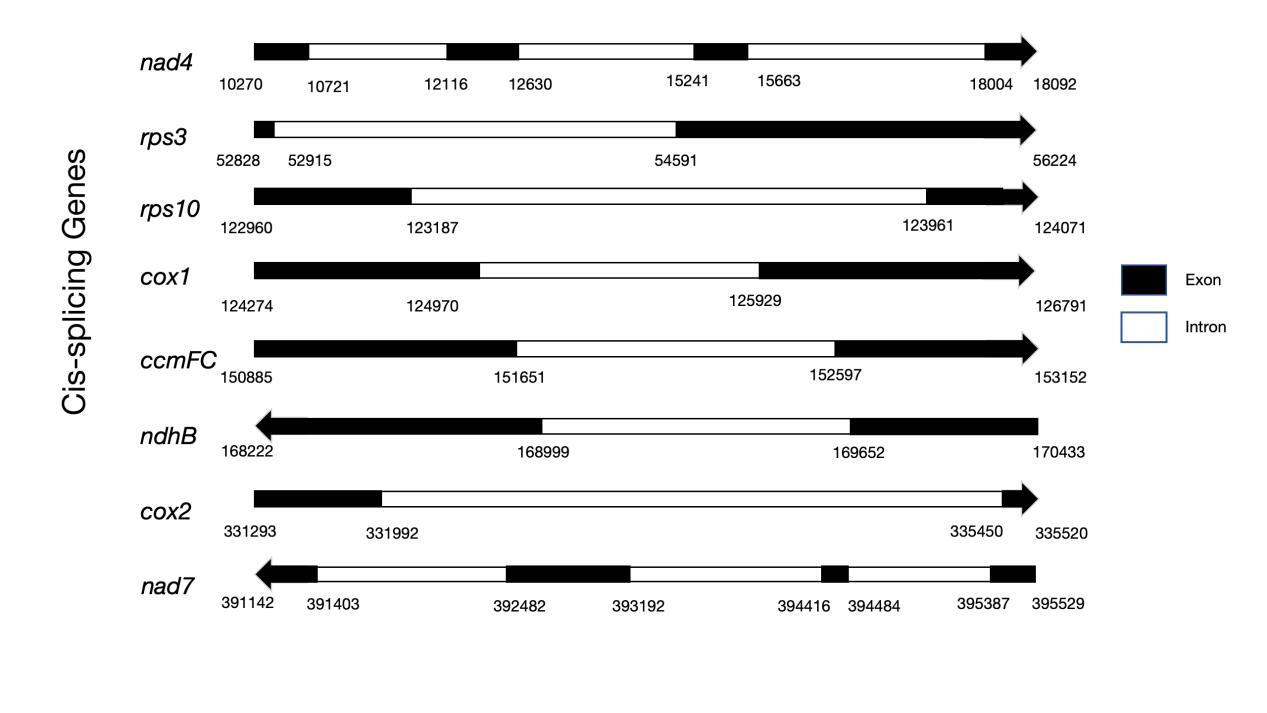


Figures S3. The schematic map illustrates the arrangement of cis-splicing genes within the R. glutinosa genome, with exons and introns depicted in black and white, respectively. The directionality of the genes is indicated by arrows.


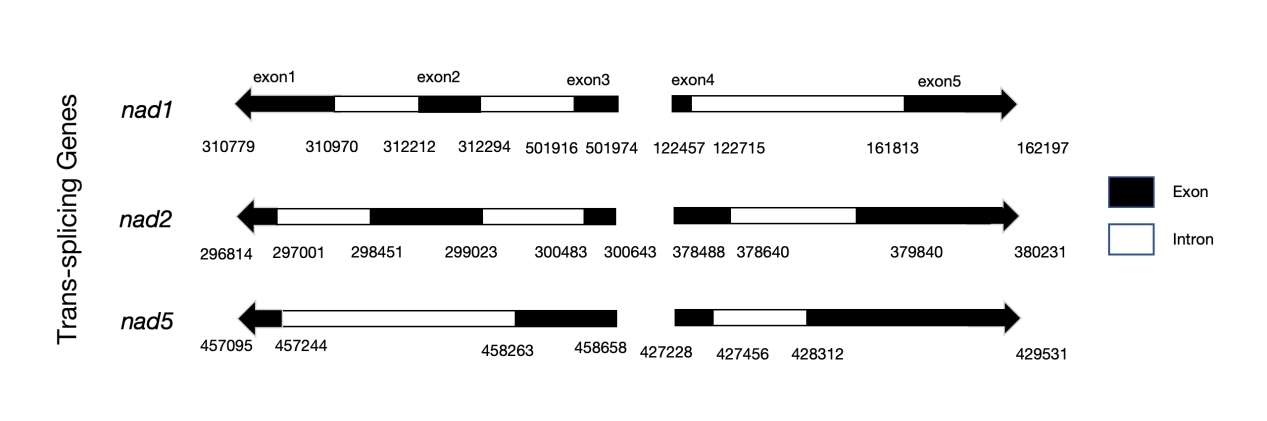


Figures S4. The schematic map illustrates the arrangement of trans-splicing genes within the R. glutinosa genome, with exons and introns depicted in black and white, respectively. The directionality of the genes is indicated by arrows.
