# Supplementary material for: The complete mitochondrial genome of an important medicinal plant, Rehmannia glutinosa (Gaertn.) DC., 1845 (Lamiales, Orobanchaceae)
Source: Mitochondrial DNA B Resour. 2024 Dec 21;10(1):21–5. doi: 10.1080/23802359.2024.2444611 (PMC11703138; doi:10.1080/23802359.2024.2444611)
Supplement: （The clen copy）Figure.docx [file TMDN_A_2444611_SM2891.docx]

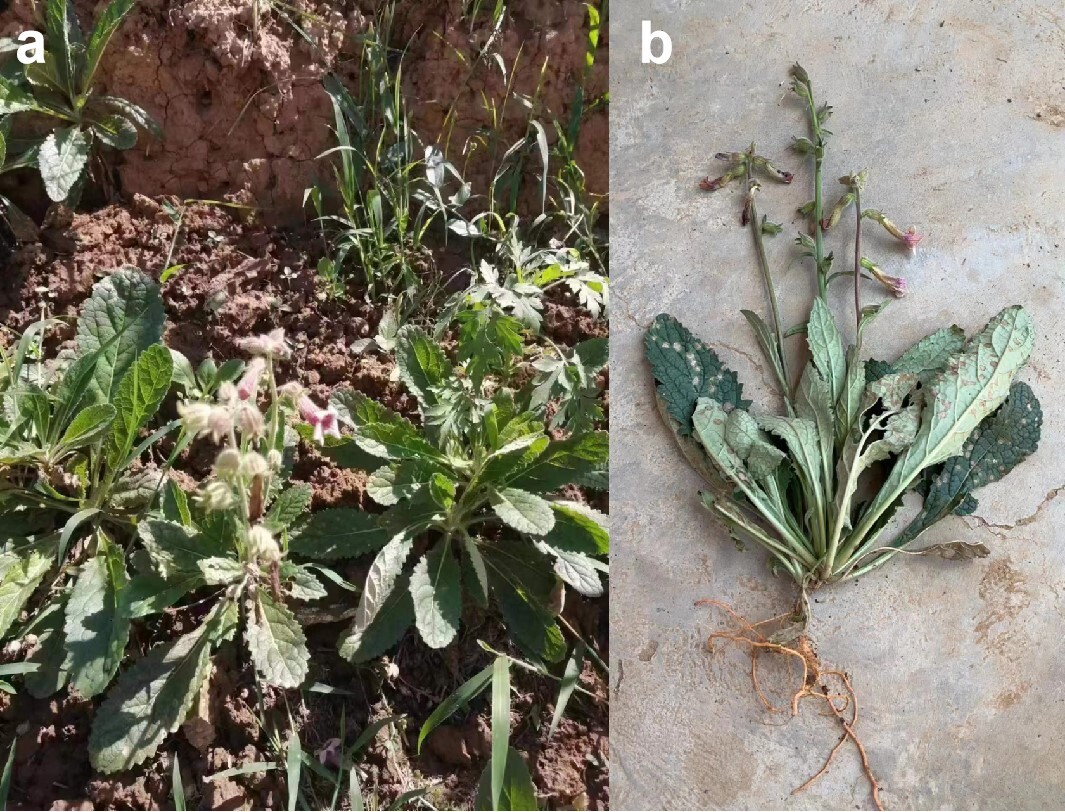


**Figure 1. Morphology characteristics of *R. glutinosa* during flowering.** (a) Basal leaves are usually rosulate. Stem leaves gradually decrease in size or are reduced to bracts upwards; leaf blades are ovate to narrowly elliptic; margins are irregularly crenate or obtusely serrate to toothed. Flowers are axillary or in terminal racemes. Pedicels are slender and ascending. (b) The fresh roots of *R. glutinosa* are yellow and can be used in traditional Chinese medicine. Photographs of *R. glutinosa* were taken by Huan An in Yuanshi County, Hebei Province, China (N 37.73°, E 114.51°) .

**
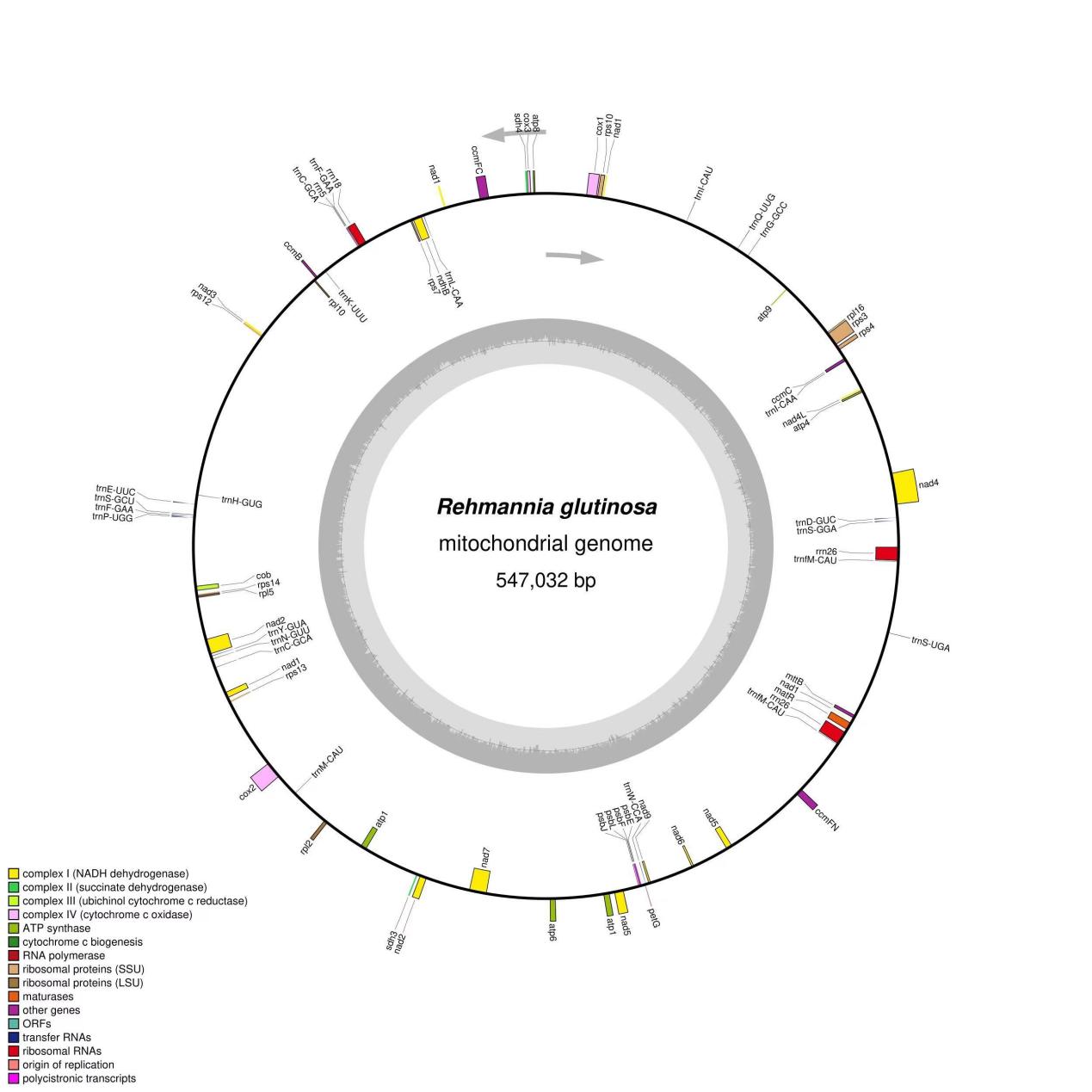
**

**Figure 2. The gene map for the complete mitochondrial genome of *R. glutinosa*.** Genes in the inner circle are transcribed clockwise, while those in the outer circle are transcribed counterclockwise. Different functional groups of genes are color coded. Darker gray shading represents DNA G + C content, while the lighter gray corresponds to A + T content.The functional classification appears in the bottom left corner.

**
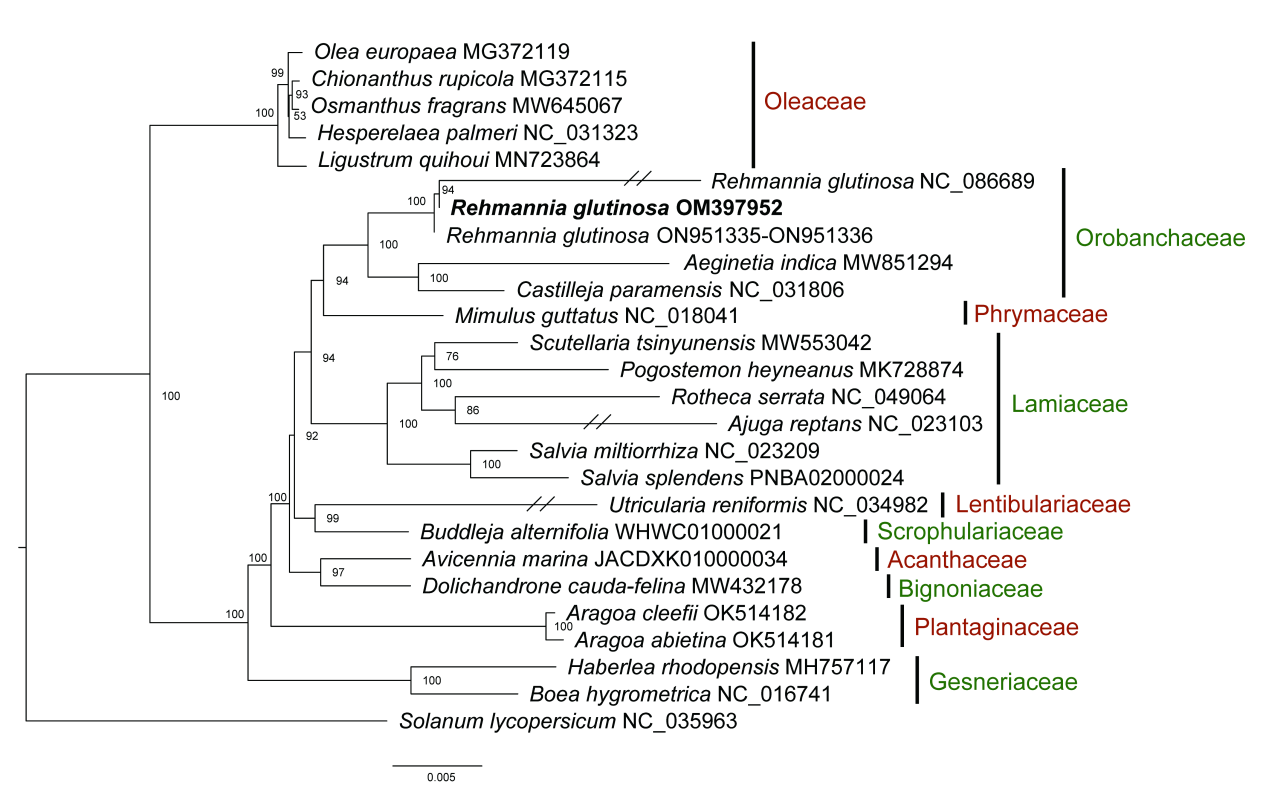
**

**Figure 3**. **Phylogenetic tree includes *R. glutinosa* and its 23 relatives, highlighting the position of *R. glutinosa* (OM397952) in bold.** A Maximum-Likelihood method was used to conduct the tree from a concatenated alignment of 37 mitochondrial protein-coding genes using GTR+F+R3 model with 1000 bootstrap replicates. The following sequences were used: *Rehmannia glutinosa* ON951335-ON951336 (Zeng et al., 2024), *Rehmannia glutinosa* NC_086689, *Olea europea* MG372119 (Van de Paer et al., 2018), *Chionanthus rupicola* MG372115 (Van de Paer et al., 2018), *Osmanthus fragrants* MW645067 (Wang & Zhang, 2021), *Hesperelaea palmeri* NC_031323 (Van de Paer et al., 2016), *Ligustrum quihoui* MN723864, *Aeginetia indica* MW851294 (Choi & Park, 2021), *Castilleja paramensis* NC_031806, *Mimulus guttatus* NC_018041 (Mower et al., 2012), *Scutellaria tsinyunensis* MW553042(Li et al., 2021), *Pogostemon heyneanus* MK728874, *Rotheca serrata* NC_049064, *Ajuga reptans* NC_023103(Zhu et al., 2014), *Salvia miltiorrhiza* NC_023209, *Salvia splendens* PNBA02000024 (Jia et al., 2021), *Utricularia reniformis* NC_034982(Silva et al., 2017), *Buddleja alternifolia* WHWC01000021 (Ma et al., 2021), *Avicennia marina* JACDXK010000034, *Dolichandrone cauda-felina* MW432178, *Aragoa cleefii* OK514182 (Mower et al., 2021), *Aragoa abietina* OK514181 (Mower et al., 2021), *Haberlea rhodopensis* MH757117, *Boea hygrometrica* NC_016741 (Zhang et al., 2011), *Solanum lycopersicum* NC_035963. *Solanum lycopersicum* (Solanales) was set as the outgroup. The branches of *Ajuga reptans* and *Utricularia reniformis* were truncated since they were too long. Bootstrap support percent values are given at the nodes. Bar, 0.005 substitutions per site.
